# Supplementary material for: Sustainable use of longan seed waste as a natural coagulant aid for low-cost and eco-friendly water treatment
Source: PLoS One. 2026 Jan 28;21(1):e0340397. doi: 10.1371/journal.pone.0340397 (PMC12851455; doi:10.1371/journal.pone.0340397)
Supplement: Attachment — Submitted filename: Response to Reviewers.docx [file pone.0340397.s002.docx]

# Response to Reviewers

Manuscript ID: PONE-D-25-41229

Title: Sustainable Use of Longan Seed Waste as a Natural Coagulant Aid for Low-Cost and Eco-Friendly Water Treatment

Authors: Anuwat Aunkham et al.

**Dear Academic Editor and Reviewers,**

We sincerely appreciate the valuable comments and constructive feedback from the Academic Editor and both reviewers. All comments have been carefully addressed, and corresponding revisions were incorporated into the revised manuscript. Below is a detailed, point-by-point response describing how each issue has been handled.

## Response to Reviewer #1

****Comment:** Abstract - Lack of emphasis on the research gap.**

**Response:** We appreciate this comment. We have revised the abstract to highlight the limitations of conventional alum-based coagulation and the motivation for using longan seed waste. (Page 1, Lines 5–12)

****Comment:** Abstract - Novelty unclear.**

**Response:** We clarified the novelty of the study by emphasizing the first assessment of longan seed powder as a coagulant aid with alum and its cost and environmental advantages. (Page 1, Lines 13–20)

****Comment:** Introduction - Need explicit research gaps.**

**Response:** We added a paragraph specifying that limited studies have explored the combined use of longan seed powder and alum for both synthetic and natural water, highlighting this research gap. (Page 3, Lines 60–70)

****Comment:** Methods - Lack of replication and control details.**

**Response:** We added the number of replicates (n=3), details of control setups, and standard deviations for all experiments. (Page 6, Lines 150–160)

****Comment:** FTIR and SEM insufficiently detailed.**

**Response:** We revised the Materials and Methods to include FT-IR resolution (4 cm⁻¹, 400–4000 cm⁻¹), SEM voltage (15 kV), and sample preparation details. (Page 8, Lines 210–220)

****Comment:** Results and Discussion - Missing graphs and comparisons.**

**Response:** We added figures and a new comparative table summarizing results from previous studies versus this work. (Page 11–12)

****Comment:** Conclusion - Lack of quantitative summary.**

**Response:** We included key quantitative outcomes (turbidity removal 95.6 ± 1.8%, TDS reduction 82.3 ± 3.2%) and linked them to alum dosage reduction by fivefold. (Page 18, Lines 12–25)

****Comment:** Conclusion - Missing limitations and future work.**

**Response:** We added a paragraph describing the limitation to lab-scale experiments and proposed future field-scale validation and biodegradability assessment. (Page 18, Lines 26–35)

## Response to Reviewer #2

****Comment:** Why longan seed compared to other starch sources?**

**Response:** We clarified in the Introduction that longan seed waste is abundant in northern Thailand (approximately 20,000 tons annually) and contains high polysaccharide and tannin content beneficial for coagulation. (Page 3, Lines 65–80)

****Comment:** Eco-friendly aspect needs support.**

**Response:** We added discussion on environmental advantages, including reduced alum sludge, biodegradable organic residues, and potential reuse of sludge as fertilizer. (Page 14, Lines 290–310)

****Comment:** Extraction and bioactive compounds unclear.**

**Response:** We clarified that longan seed powder was used as crude form, primarily containing starch and polyphenols. We also discussed this limitation and future identification of bioactive components. (Page 7, Lines 170–190)

****Comment:** No sample size information.**

**Response:** We added explicit mention that each jar test was conducted in triplicate (n=3) with statistical analysis (ANOVA, p<0.05). (Page 6, Lines 150–160)

****Comment:** SEM conditions missing.**

**Response:** We added SEM imaging details: JEOL JSM-6480LV, acceleration voltage 15 kV, gold-coated samples. (Page 8, Lines 210–215)

****Comment:** Mechanism discussion incomplete.**

**Response:** We expanded the discussion by linking FT-IR peaks (–OH, C–O–C) to charge neutralization and bridging effects. (Page 16, Lines 340–355)

****Comment:** Plant extract stability not addressed.**

**Response:** We included information about storage conditions (airtight, room temperature, ≤30°C) and 6-month stability observation. (Page 7, Lines 195–205)

****Comment:** Cost comparison oversimplified.**

**Response:** We added clarification that production cost estimation excludes logistics and scaling factors, which are proposed for future techno-economic analysis. (Page 17, Lines 365–380)

****Comment:** Add final statement in conclusion.**

**Response:** We added a concise end paragraph highlighting how this study supports sustainable water treatment and provides a foundation for eco-friendly coagulant development. (Page 18, Lines 32–38)

## Response to the Academic Editor and Journal Requirements

****Comment:** Formatting compliance**

**Response:** The manuscript has been formatted according to the PLOS ONE template for both title/author page and main body.

****Comment:** Permits and ethical approval**

**Response:** The study involved the collection of water samples from public natural water sources located in Chiang Rai Province, Thailand. These water bodies are under the supervision of the Chiang Rai Provincial Administrative Organization (PAO). According to Thai environmental regulations, no specific sampling permit is required for collecting water from public natural sources, as they are open-access and available for general community use. Nevertheless, all sampling activities were conducted responsibly and under the approval and supervision of the Environmental Health Program, School of Health Science, Mae Fah Luang University. (Page 5, Lines 120–135)

****Comment:** Data availability**

**Response:** All raw and processed data have been included within the manuscript and supplementary files, following PLOS ONE data policy.

****Comment:** Funding statement**

**Response:** All funding information has been moved exclusively to the Funding Statement section as required.

****Comment:** Authorship update**

**Response:** All co-authors, including Budsakorn Aiyee, Ploypairin Choochan, Phitchayapha Chumueang, Wichuda Intawong, Natcha Thambun, Anantaya Anantaburi, and Panadda Klinchan, have been added to the submission system.

****Comment:** Figure 3 reference**

**Response:** We inserted an in-text reference to Figure 3 in the Results section. (Page 12, Line 260)

We hope that these revisions meet the expectations of the reviewers and the Academic Editor. We believe that the revised manuscript has been substantially improved in clarity, methodological rigor, and overall contribution to sustainable water treatment research.

**Sincerely,
Anuwat Aunkham (on behalf of all authors)**
