# Supplementary material for: Sustainable use of longan seed waste as a natural coagulant aid for low-cost and eco-friendly water treatment
Source: PLoS One. 2026 Jan 28;21(1):e0340397. doi: 10.1371/journal.pone.0340397 (PMC12851455; doi:10.1371/journal.pone.0340397)
Supplement: Attachment — Submitted filename: Response to Reviewers-2.docx [file pone.0340397.s003.docx]

**Response to Reviewers**

**Manuscript ID:** PONE-D-25-41229R1

**Title:** *Sustainable Use of Longan Seed Waste as a Natural Coagulant Aid for Low-Cost and Eco-Friendly Water Treatment*

**Authors:** Anuwat Aunkham et al.

Dear Academic Editor and Reviewers,

We sincerely thank the Academic Editor and both reviewers for the insightful and constructive comments provided on our manuscript. We have carefully revised the manuscript to address **every comment in full**, ensuring that no point was overlooked. Below, we provide a comprehensive point-by-point response detailing how each comment has been addressed in the revised version.

**Response to Reviewer #1**

**Abstract**

**Comment 1: Lack of emphasis on the gap or problem to be solved.**

**Response:** The abstract has been revised to clearly emphasize the environmental and operational limitations of conventional alum-based coagulation and the motivation for exploring longan seed waste as a sustainable alternative. *(Page 2, Lines 22–31)*

**Comment 2: Novelty of the research is unclear.**

**Response:** We now explicitly highlight that this study provides the first systematic evaluation of longan seed powder (LSP) as a coagulant aid with alum in both synthetic and natural waters, including cost advantages and environmental relevance. *(Page 2, Lines 32–43)*

**Comment 3: Lack of information on experimental design and replication.**

**Response:** The revised abstract now includes information on replication (n = 3), control conditions, and experimental scale. *(Page 2, Lines 24–27)*

**Comment 4: Insufficient emphasis on environmental or practical implications.**

**Response:** We added statements clarifying the real-world applicability of LSP in reducing alum sludge and providing low-cost treatment options. *(Page 2, Lines 38–42)*

**Comment 5: Lack of limitations or further research recommendations.**

**Response:** A concluding sentence has been added summarizing the study limitations and suggesting pilot-scale and biodegradability investigations. *(Page 2, Lines 41–43)*

**Introduction**

**Comment 6: Research gaps not explicitly stated.**

**Response:** A new paragraph now explicitly addresses the research gaps, noting the absence of systematic evaluations of LSP–alum combinations in both synthetic and natural waters. *(Page 3, Lines 60–70)*

**Comment 7: Novelty and uniqueness unclear.**

**Response:** We clarified the novelty in terms of minimal alum dosage, effects on TDS/pH, and comparison to prior coagulant-aid studies. *(Page 3, Lines 72–80)*

**Comment 8: Insufficient quantitative background (e.g., longan waste volume).**

**Response:** We added global and Asian production data (>3 million tons annually) to demonstrate scalability and availability of longan seed waste. *(Page 4, Lines 80–85)*

**Comment 9: Weak linkage between coagulation theory and longan seed chemistry.**

**Response:** We included new text explaining how polysaccharide-rich biopolymers in longan seeds support adsorption and bridging mechanisms. *(Page 4, Lines 86–95)*

**Comment 10: Environmental and economic context insufficient.**

**Response:** We expanded the introduction to include cost advantages, waste valorization, and reduced alum sludge generation. *(Page 4, Lines 96–102)*

**Comment 11: Relevance to real-world application unclear.**

**Response:** New text discusses applicability in rural water supply systems and decentralized water treatment. *(Page 4, Lines 103–108)*

**Materials and Methods**

**Comment 12: Lack of replication details and control conditions.**

**Response:** Added explicit replication (n = 3), negative controls (no coagulant), and positive controls (alum only). *(Page 5, Lines 108–115)*

**Comment 13: Environmental conditions missing (temperature, humidity).**

**Response:** Added environmental conditions (25 ± 2 °C, humidity 65 ± 5%). *(Page 5, Lines 106–108)*

**Comment 14: Statistical analysis not fully described.**

**Response:** Methods now include details on ANOVA, confidence levels (p < 0.05), and use of standard deviations. *(Page 7, Lines 160–168)*

**Comment 15: Insufficient FTIR and SEM measurement information.**

**Response:** FTIR parameters (4 cm⁻¹ resolution, 400–4000 cm⁻¹ range) and SEM conditions (JEOL JSM-6480LV, 15 kV, gold coating) have been added. *(Page 6, Lines 134–145)*

**Comment 16: Missing kaolin concentration and initial pH conditions.**

**Response:** We added kaolin dosages used to generate NTU levels and details on initial pH control. *(Page 5, Lines 110–118)*

**Comment 17: Longan seed powder preparation details insufficient.**

**Response:** Added particle size distribution after grinding and sieve mesh specifications. *(Page 5, Lines 119–124)*

**Comment 18: TDS and pH measurement details missing.**

**Response:** Added calibration procedures and measurement standards. *(Page 7, Lines 164–168)*

**Comment 19: Jar-test mixing method unclear.**

**Response:** Added details on paddle configuration, rotational uniformity, and hydrodynamic control. *(Page 5, Lines 112–118)*

**Comment 20: Lack of information on reproducibility and scalability.**

**Response:** A new subsection discusses potential scalability and reproducibility considerations. *(Page 8, Lines 190–195)*

**Comment 21: Missing integration between chemical characterization and coagulation performance.**

**Response:** Clarified cross-references linking FTIR/SEM results to observed coagulation efficiency. *(Page 16, Lines 340–355)*

**Comment 22: Reviewer requested sketches for experiment setup.**

**Response:** Added schematic diagrams in the Supplementary Materials. *(Supplementary)*

**Results and Discussion**

**Comment 23: Results should include graphs.**

**Response:** Multiple figures (turbidity removal, TDS, particle size, dosage response) have been added. *(Page 11–13, Lines Various)*

**Comment 24: FTIR and SEM results missing.**

**Response:** FTIR spectra and SEM micrographs are now included with detailed interpretation. *(Page 14–15, Lines 300-335)*

**Comment 25: Discussion insufficient; add comparative table.**

**Response:** Added a comparison table with previous studies and expanded the discussion to analyze similarities and differences. *(Page 15, Table 3)*

**Conclusion**

**Comment 26: Lack of quantitative results in conclusion.**

**Response:** Added mean turbidity removal (95.6 ± 1.8%) and TDS reduction values. *(Page 18, Lines 390-395)*

**Comment 27: Limitations not adequately addressed.**

**Response:** Added limitations regarding laboratory scale and variability in seed composition. *(Page 18, Lines 396-402)*

**Comment 28: Mechanism not linked clearly to results.**

**Response:** Added final synthesis connecting FTIR/SEM findings to charge neutralization and polymer bridging. *(Page 18, Lines 403-407)*

**Comment 29: Environmental/social implications missing.**

**Response:** Included implications such as reduced alum sludge and valorization of agricultural waste. *(Page 18, Lines 408-412)*

**Comment 30: No mention of research generalizability.**

**Response:** Added explanation on applicability to river water, domestic water, and similar systems. *(Page 18, Lines 413-416)*

**Comment 31: Contribution of the research not highlighted.**

**Response:** Added statements summarizing scientific and practical contributions. *(Page 18, Lines 417-420)*

**Response to Reviewer #2**

**Comment 32: Why use longan seed compared to other starch sources?**

**Response:** Added global and national data showing abundance of longan seed waste and its polysaccharide/tannin composition advantages. *(Page 3-4, Lines 74-85)*

**Comment 33: Eco-friendly claim needs experimental support.**

**Response:** Expanded discussion on reduced alum sludge, biodegradability expectations, and future toxicity evaluation needs. *(Page 14, Lines 292-305)*

**Comment 34: Need detail on compounds in crude extract.**

**Response:** Clarified crude composition (polysaccharides, polyphenols) and acknowledged that further compound-level analysis is planned. *(Page 7, Lines 170-180)*

**Comment 35: Replication/sample size not clear.**

**Response:** Now explicitly detailed (n = 3) with ANOVA statistical validation. *(Page 35, Lines 160-168)*

**Comment 36: SEM measurement conditions missing.**

**Response:** Included full details of SEM settings and sample preparation. *(Page 6, Lines 134-145)*

**Comment 37: Coagulation mechanism not fully demonstrated.**

**Response:** Added dosage-response plots and mechanism-linked interpretation supported by FTIR/SEM. *(Page 13-16, Lines 270-355)*

**Comment 38: Stability of plant extracts.**

**Response:** Added a subsection describing storage conditions and stability observations over six months. *(Page 7, Lines 181-188)*

**Comment 39: Cost comparison oversimplified.**

**Response:** Added statements acknowledging scaling and logistics constraints and noted as future techno-economic analysis. *(Page 7, Lines 365-380)*

**Comment 40: Add final summary statement.**

**Response:** The conclusion now ends with a strengthened summary paragraph highlighting major outcomes and future directions. *(Page 7, Lines 417-420)*

**Response to Academic Editor Requirements**

**Comment 41: Formatting compliance.**

**Response:** Manuscript reformatted according to the PLOS ONE template for both title and main body. *(All)*

**Comment 42: Permits and fieldwork authorization.**

**Response:** Added statement clarifying permit requirements and institutional approvals. *(Page 5, Lines 120-135)*

**Comment 43: Data availability.**

**Response:** Updated Data Availability Statement and ensured all data are provided in manuscript and Supplementary files. *(Page 9, Data section)*

**Comment 44: Funding statement correction.**

**Response:** Removed all funding information from the manuscript body and retained it only in the official Funding Statement. *(Submission)*

**Comment 45: Authorship update.**

**Response:** Added all required co-authors to the manuscript and submission system.

**Comment 46: Figure 3 reference missing.**

**Response:** Now cited in the appropriate Results subsection. *(Page 12, Lines 255-260)*

We believe that these substantial revisions have significantly improved the clarity, rigor, and scientific contribution of the manuscript. We thank the reviewers again for their detailed feedback and hope the revised manuscript will now meet the journal’s publication standards.

**Sincerely,**
Anuwat Aunkham (on behalf of all authors)
